# Effects of Cathode Location and the Size of Anode on Anodal Transcranial Direct Current Stimulation Over the Leg Motor Area in Healthy Humans

**DOI:** 10.3389/fnins.2018.00443

**Published:** 2018-07-04

**Authors:** Águida S. Foerster, Zeynab Rezaee, Walter Paulus, Michael A. Nitsche, Anirban Dutta

**Affiliations:** ^1^Department of Clinical Neurophysiology, Universitätsmedizin Göttingen, Georg-August-Universität Göttingen, Göttingen, Germany; ^2^IfADo – Leibniz Research Center for Working Environment and Human Factors, Dortmund, Germany; ^3^Department of Biomedical Engineering, University at Buffalo, The State University of New York (SUNY), Buffalo, NY, United States; ^4^Department of Neurology, BG University Hospital Bergmannsheil, Bochum, Germany

**Keywords:** lower limb motor cortex, stimulation parameters, motor cortex excitability, modeling, transcranial direct current stimulation (tDCS)

## Abstract

**Objective:** Non-invasive brain stimulation such as transcranial direct current stimulation (tDCS) involves passing low currents through the brain and is a promising tool for the modulation of cortical excitability. In this study, we investigated the effects of cathode location and the size of anode for anodal tDCS of the right-leg area of the motor cortex, which is challenging due to its depth and orientation in the inter-hemispheric fissure.

**Methods:** We first computationally investigated the effects of cathode location and the size of the anode to find the best montage for specificity of stimulation effects for the targeted leg motor area using finite element analysis (FEA). We then compared the best electrode montage found from FEA with the conventional montage (contralateral supraorbital cathode) via neurophysiological testing of both, the targeted as well as the contralateral leg motor area.

**Results:** The conventional anodal tDCS electrode montage for leg motor cortex stimulation using a large-anode (5 cm × 7 cm, current strength 2 mA) affected the contralateral side more strongly in both the FEA and the neurophysiological testing when compared to other electrode montages. A small-anode (3.5 cm × 1 cm at 0.2 mA) with the same current density at the electrode surface and identical contralateral supraorbital cathode placement improved specificity. The best cathode location for the small-anode in terms of specificity for anodal tDCS of the right-leg motor area was T7 (10–10 EEG system).

**Conclusion:** A small-anode (3.5 cm × 1 cm) with the same current density at the electrode surface as a large-anode (5 cm × 7 cm) resulted in similar cortical excitability alterations of the targeted leg motor cortex respresentation. In relation to the other stimulation conditions, the small-anode montage with the cathode positioned at T7 resulted in the best specificity.

## Introduction

Clinical applications of non-invasive brain stimulation (NIBS) are currently an evolving area and increasingly used as an adjuvant treatment during motor rehabilitation (Flöel, 2014). Transcranial direct current stimulation (tDCS) is a NIBS modality that involves application of low intensity direct currents using two or more electrodes for a certain duration, which can alter corticospinal excitability polarity-dependently for up to 60 min after the end of the stimulation (Bailey et al., 2016). The first studies were conducted in the hand area of the motor cortex that showed corticospinal excitability alterations, monitored by transcranial magnetic stimulation (TMS)-induced motor evoked potentials (MEP) (Rossi et al., 2009), of up to 40%. In the motor cortex, excitability enhancement was achieved by anodal stimulation, whereas cathodal stimulation reduced excitability (Nitsche and Paulus, 2000). Moreover, the strength and duration of these after-effects are controlled by current intensity and duration (Nitsche and Paulus, 2001; Nitsche et al., 2003b; Monte-Silva et al., 2010, 2013; Batsikadze et al., 2013). Pharmacological studies (Liebetanz et al., 2002; Nitsche et al., 2003a) identified a role of tDCS-induced membrane polarization and NMDA receptor activation for these sustained after-effects (Nitsche and Paulus, 2001).

Awareness about the relevance of computational modeling for rational design of electrode montages, taking into account not only the electric field strength but also the current flow direction in relation to neuronal orientation (Das et al., 2016), has increased recently. Computational modeling can help to identify optimal electrode positions, and improve efficacy of stimulation (Datta et al., 2011). In this study, we focused on the application of tDCS over the leg area of the motor cortex, which presents a challenge due to its depth and orientation in the inter-hemispheric fissure, and has not been explored as much as tDCS of the hand area of the motor cortex. Some studies, however, have shown that tDCS can modulate the excitability of the leg area of the motor cortex. Jeffery et al. (2007) showed that 10 min of stimulation with the anode over the leg area of the motor cortex in healthy humans increased corticospinal excitability of the anterior tibial (TA) muscle by up to 59% compared to baseline values for up to 60 min after stimulation. Cathodal tDCS, however, did not decrease corticospinal excitability. In a functional outcome study in healthy humans, anodal tDCS has been shown to transiently enhance maximal leg pinch force for up to 30 min after stimulation compared to baseline, but did not affect reaction time (Tanaka et al., 2009). Also here, cathodal tDCS did not alter performance. Roche et al. (2011) showed that anodal tDCS over the same area induced effects on spinal network excitability similar to those observed during co-contraction of lower-limb muscles. Such indirect effects on spinal network excitability may be suited to support postural stability and balance, as shown by the recent studies conducted in healthy humans (Dutta et al., 2014a; Kaminski et al., 2016).

Regarding clinical application of tDCS over the primary motor cortex leg area, anodal stimulation on the lesioned cortex with a large square sponge electrode (5 cm × 5 cm) with 2 mA for 10 min improved balance and strengthened the affected lower limb in stroke patients (Sohn et al., 2013). Jayaram and Stinear (2009) explored the effects of anodal tDCS over the lesioned motor cortex of nine chronic stroke survivors using a small 8.1 cm^2^ saline-soaked sponge electrode as anode (unlike most other studies, which used relatively large 25–36 cm^2^ stimulation electrodes) whose edge was aligned to the midsagittal plane, and a large 36 cm^2^ cathode which was placed above the contralateral orbit. They investigated bilateral modulatory effects of stimulation on the tibialis anterior (TA), medial gastrocnemius, medial hamstrings, and vastus lateralis muscles. Anodal tDCS over the ipsilesional motor cortex increased paretic limb and decreased non-paretic limb motor excitability, and thus showed a relatively focal effect. Regarding effects on motor functions, a single session of anodal tDCS of the paretic lower limb was shown to increase knee extensor force for up to 30 min following stimulation in hemiparetic stroke survivors (Tanaka et al., 2011). van Asseldonk and Boonstra (2016) showed similar montage-related performance differences in 10 healthy subjects and 10 chronic stroke survivors that also revealed a large inter-individual variability of effects. In that study, two montages with a 5 cm × 7 cm anode placed over the lesioned hemisphere with the short edge of the rectangular electrode aligned to the mid-sagittal fissure and centered over the motor cortex representation of the leg, and the cathode placed over the supraorbital region (called unihemispheric montage) or over the motor cortex contralateral to the targeted area (called bihemispheric montage) were compared. In the study of van Asseldonk and Boonstra (2016), subjects with the largest effect for one montage often showed opposite effects for the other. This underscores the relevance of the placement of the electrodes when aiming to stimulate the leg area, analogous to what has been described for the hand area (Bikson et al., 2010; Moliadze et al., 2010). Placement of the electrodes is not only critical for the electric field strength, but also electric field direction (Rawji et al., 2018). Both factors are relevant for stimulation of the leg area of the motor cortex due to its depth and orientation in the inter-hemispheric fissure. However, a comprehensive finite element modeling of tDCS of the leg motor area with a realistic head model and physiological validation of the computational results has not been conducted so far. Stimulation parameters and brain anatomy affect efficacy and specificity of tDCS, which is particularly challenging for cortical areas not on the brain surface such as the leg area of the motor cortex.

In our preliminary study (Dutta et al., 2012) using a simple three-shell head model, we hypothesized that not only the electric field strength but also the electric field direction is relevant for the effects of anodal tDCS over the leg motor area. For the present study, our goal was to maximally stimulate the targeted leg motor representation while avoiding stimulation of the contralateral leg motor volume. We investigated simple two-electrode unihemispheric montages using a realistic computational head model and explored the impact of cathode placement and anode size on anodal tDCS over the motor cortex leg area. We then evaluated the appropriateness of the computational models via neurophysiological testing in healthy individuals.

## Materials and Methods

### Finite Element Model of the Human Head

The head model for finite element modeling was developed using the freely available SimNIBS software pipeline.^1^ The SimNIBS software pipeline (Windhoff et al., 2013) uses fat-suppressed T1-weighted magnetic resonance images (MRI) as input for FreeSurfer (Fischl, 2012). We used the Colin27 average brain (Collins et al., 1998; Holmes et al., 1998), which is the stereotaxic average of 27 T1-weighted MRI scans of the same individual, to create the head model (see iso2mesh toolbox (iso2mesh; Fang and Boas, 2009). The Colin27 average brain has been widely adopted as a stereotaxic template that includes and labels cerebellum, brain stem, and ventricles. After segmentation, different components like scalp, skull, cerebrospinal fluid (CSF), white matter (WM), and gray matter (GM) of the brain were modeled as different volume conductors with their own specific conductivity (Windhoff et al., 2013), as shown in **Table 1**.

**Table 1:** Electrical conductivity.

Component	Electrical conductivity (S m^-1^)
Scalp	0.465
Skull	0.010
CSF	1.654
Gray matter	0.276
White matter	0.126


The anode and cathode injected a specified amount of current (source) in the volume conductor. The electrodes were modeled as saline-soaked sponge cuboids (see section “Electrode Montages for Finite Element Modeling”). We analyzed the head-model for electric field distribution using the Finite Element Method (FEM), provided in the SimNIBS pipeline, which provides a powerful numerical tool to solve the required partial differential equations (PDE).

**FIGURE 1:**
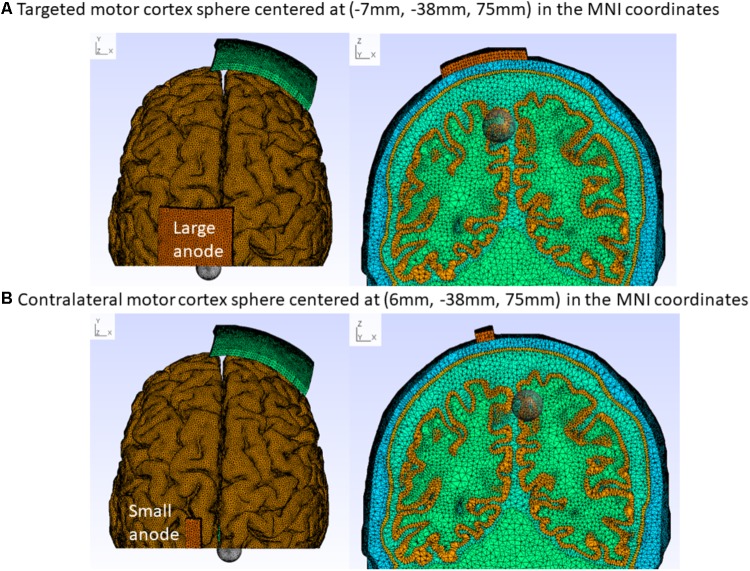
Colin27 FEM head model for the “conventional montage” electrode arrangement with the cathode (5 cm × 7 cm) at Fp2 (10–10 EEG system) and anode centered at 15 mm left lateral and 20 mm posterior to Cz (10–10 EEG system). **(A)** Large-anode: 5 cm × 7 cm at 2 mA, **(B)** Small-anode: 3.5 cm × 1 cm at 0.2 mA. The cortical tissue cluster found after Boolean intersection with the sphere of a radius of 1 cm captures the electric field at the targeted [centered at (–7 mm, –38 mm, 75 mm) MNI coordinates – **A**] and the contralateral [centered at (6 mm, –38 mm, 75 mm) MNI coordinates – **B**] leg motor cortex, as shown with top (left panel) and coronal (right panel) sectional views.

The quasi-static formula for direct current stimulation is given below,

−∇⋅(σ∇V)=S in Ω

where Ω is the volume conductor, *V*_(*x,y,z*)_ is the scalar potential field, σ_(*x,y,z*)_ is the conductivity tensor, *S* is the source term. The Dirichlet boundary condition is presented in Section “Electrode Montages for Finite Element Modeling”. FEM divides the volume conductor into spatial elements and nodes for discrete computations of the PDE. The tetrahedral head meshes for FEM were generated using the “mri2mesh” tool in the SimNIBS software pipeline (Windhoff et al., 2013) with an average tetrahedron volume of 1 mm^3^. The continuity of the solution is maintained at the boundary of the elements using shape function objects. The electric field values at the nodes within the bilateral leg area cluster in the cortical tissue (not CSF) were captured by Boolean intersection with a sphere of 1 cm radius centered at (-7 mm, -38 mm, 75 mm) and (6 mm, -38 mm, 75 mm) in the MNI coordinates, as shown in **Figure 1**. The cortical tissue cluster after Boolean intersection with the sphere was comparable to the functional MRI activation volume (∼1450 mm^3^) during plantar (45°) and dorsal flexion (10°) of the foot at a rate of approximately 0.5 Hz (Alkadhi et al., 2002). All node values within the cortical tissue clusters were imported in Matlab (The Mathworks, Inc., United States) to compute the average magnitude and direction (described in section “Optimization of Electrode Montage”).

### Electrode Montages for Finite Element Modeling

The electrode positions were defined with fiducials at Nz, Iz, right, and left preauricular points for registration with the head model in accordance with the 10–10 system defined in Oostenveld and Praamstra (2001). We explored the effects of the following electrode positions, and sizes: motor cortex anode (large: 5 cm × 7 cm at 2 mA and small: 3.5 cm× 1 cm at 0.2 mA) at the approximate TA muscle hotspot based on neurophysiological testing (Dutta et al., 2014b) – 15 mm left lateral and 20 mm posterior to Cz (1 mm, -28 mm, 87 mm). The cathode (5 cm × 7 cm) was placed at Fp1(-21 mm, 70 mm, 15 mm), F7(-53 mm, 32 mm, 2 mm), T7(-70 mm, -16 mm, -8 mm), P7(-58 mm, -65 mm, -6 mm), Oz(1 mm, -101 mm, 6 mm), P8(56 mm, -64 mm, -6 mm), T8(55 mm, 30 mm, -1 mm), and Fp2(25 mm, 68 mm, 15 mm). Here, (*x*, *y*, and *z*) refer to the MNI stereotaxic space (Jurcak et al., 2007); the *x* direction is medio-lateral, the *y* direction anterior-posterior, and the *z* direction ventro-dorsal. This resulted in eight montages with cortical projection of their respective electrode center denoted using the 10–10 EEG system (Koessler et al., 2009). The contralateral supraorbital cathode position (Fp2) was termed “conventional montage,” since this montage was most often used in prior tDCS studies of the leg motor area (Madhavan and Shah, 2012). These eight electrode montages were evaluated computationally, as described in Section “Optimization of Electrode Montage,” based on the Colin27 FEM head model (see section “Finite Element Model of the Human Head”). Transcranially injected direct current per unit area at the top of the saline-soaked sponge anode was set constant at 0.057 mA/cm^2^ which was a Dirichlet boundary condition for the FEM head model.

### Optimization of Electrode Montage

The tDCS current per unit area at the top of the sponge electrodes was kept constant at 0.057 mA/cm^2^ for the computational optimization of the electrode montage that resulted in 2 mA direct current for the large-anode and 0.2 mA for the small-anode. This current amplitude is considered to be safe and adequate for experimental validation in healthy humans (Nitsche et al., 2003b). The electric field values (see section “Finite Element Model of the Human Head”) at the nodes within the bilateral leg motor volume were extracted with the “CutSphere” command of Gmsh^2^ (Geuzaine and Remacle, 2009) and imported in Matlab (The Mathworks Inc., United States) as a text file for computing their average magnitude and direction. Here, the cortical tissue cluster found after Boolean intersection of the cortical tissue with the sphere of a radius of 1 cm with centroids at (-7 mm, -38 mm, 75 mm) and (6 mm, -38 mm, 75 mm) in MNI coordinates (see **Figure 1**) represented the targeted and contralateral leg motor volume respectively. The specificity of the electric field $\left(\underset{EF}{\to }\right)$ for different cathode locations (Fp1, F7, T7, P7, Oz, P8, T6, T8, and Fp2) was determined by the laterality of the volume-averaged electric field strength (Opitz et al., 2015) toward the targeted leg motor volume. Therefore, the specificity was computationally (comp) found based on the volume-averaged magnitude of the electric field $\left(|EF|=\sqrt{EFoEF}\right)$ or volume-averaged electric field strength (Opitz et al., 2015),

$${Specificity}_{comp}^{montage}=\frac{\left({\left|EF\right|}_{\mathrm{targeted}}-{\left|EF\right|}_{contralateral}\right)}{\left({\left|EF\right|}_{\mathrm{targeted}}+{\left|EF\right|}_{contralateral}\right)}$$

The best montage based on the computational (comp) analysis, ${Specificity}_{comp}^{montage}$, was compared with the “conventional montage” based on neurophysiological testing (see section “Experimental Validation”). Our goal was to maximally stimulate the targeted leg motor volume [centroid at (-7 mm, -38 mm, 75 mm) MNI coordinates] while avoiding stimulation of the contralateral leg motor volume [centroid at (6 mm, -38 mm, 75 mm) MNI coordinates] – see **Figure 1**. The volume-averaged electric field $\left(\underset{EF}{\to }\right)$ unit vector was also computed for the targeted (targ in Equation 2b) and contralateral (contra in Equation 2b) leg motor volumes, and the angle between these vectors was used for comparison.

$${Angle}_{comp}^{montage}={∠\overset{\to }{EF}}_{\mathrm{targeted}}-{∠\overset{\to }{EF}}_{contralateral}$$

### Experimental Validation

Twelve healthy subjects, seven males and five females (age: 21–36 years, all right-leg dominant) volunteered for the study. The subjects signed an informed consent form before participation and the study was approved by the Institutional Review Board of the University Medical Center, Goettingen, Germany. The experiment consisted of multiple sessions of anodal or sham tDCS with each session addressing a separate electrode montage (list given in Table 2, complete cross-over design) in randomized order, with sufficient (1 week) “wash-out” time in between the sessions.

**Table 2:** Electrode montages and stimulation parameters for neurophysiological testing.

Montage	Anode	Cathode
Large-anode in *conventional montage*	5 cm × 7 cm at 2 mA	5 cm × 7 cm at FP2
Small-anode in *conventional montage*	3.5 cm × 1 cm at 0.2 mA	5 cm × 7 cm at FP2
Small-anode in *side montage*	3.5 cm × 1 cm at 0.2 mA	5 cm × 7 cm at T7
Small-anode in *sham montage*	3.5 cm × 1 cm at 0 mA	5 cm × 7 cm at FP2


The anode was placed over the dominant right-leg motor cortex representation, as shown in **Figure 1**. **Figure 1** also shows the targeted and contralateral leg motor volumes, which were used to compute the specificity of the stimulation. A transcranial DC stimulator (NeuroConn, Germany) delivered the currents for 10 min via the anode centered on the scalp at the position where TMS of the primary motor cortex elicited maximal twitches in the resting dominant right-leg TA muscle. TMS was delivered with a Magpro Stimulator (MagVenture, United States) through a butterfly coil (MC-B70, MagVenture, United States) and the resting muscle activity as well as the MEP were monitored using biofeedback software (Signal 2 software, CED, United Kingdom). For TMS of the right-leg motor area, a right-to-left oriented current flow in the brain tissue is required for MEP generation and conversely, when stimulating the left-leg motor area with TMS, a left-to-right oriented current is optimal. The handle of the TMS butterfly coil was thus aligned approximately 90° to the parasagittal plane to induce a tissue current that runs in the coronal plane in the required direction (Groppa et al., 2012). The location of the coil on the scalp for the targeted right-leg, called the “target-hotspot,” was identified with single-pulse TMS by adjusting the coil position until it resulted in the largest MEP at a moderate suprathreshold stimulation intensity. Then, the contralateral left-leg hotspot, called the “contralateral hotspot,” was identified. Both hotspots were marked with water-resistant ink to reduce variability of coil placement during bilateral testing of corticospinal excitability. Corticospinal excitability alterations (Rossini et al., 1999) were evaluated using single-pulse TMS intensity that elicited 10 MEPs of average 0.5 mV amplitude at baseline before intervention. Corticospinal excitability was monitored at the “target-hotspot” as well as the “contralateral-hotspot.” Corticospinal excitability was measured before and immediately after the completion of tDCS as well as every 15 min for the next 60 min, and then every 30 min for next 60 min for each session, and 24 h for the real stimulation conditions, 10 MEPs were recorded for each time bin. For sham tDCS, the current was ramped up for 15 s and then down to zero in 15 s for blinding purposes. All subjects included in this study responded at baseline to single-pulse TMS with 10 MEPs of an average 0.5 mV at the “target-hotspot” as well as at the “contralateral-hotspot.”

During anodal tDCS of the “target-hotspot,” the current was ramped up linearly for 15 s to a constant amplitude of either 2 or 0.2 mA which was maintained for 10 min before being ramped down linearly for 15 s.

The specificity of the corticospinal excitability after-effects based on MEP-based neurophysiological (neurophys) measures at the “target-hotspot” and the “contralateral-hotspot” was computed as,

$${Specificity}_{neurophys}^{montage}=\frac{\left({MEP}_{\mathrm{targeted}}-{MEP}_{contralateral}\right)}{\left({MEP}_{\mathrm{targeted}}+{MEP}_{contralateral}\right)}$$

Here, *MEP*_targeted_ is the MEP-based measure of corticospinal excitability at the “target-hotspot” and the *MEP*_contralateral_ is the one at the “contralateral-hotspot.”

Two-way repeated measure ANOVAs (within subject factors: time post-tDCS and tDCS-condition, dependent variables: baseline-normalized MEP and ${Specificity}_{electrophys}^{montage}$) were conducted to calculate the effect of the tDCS-conditions: large-anode in the “conventional montage,” small-anode in the “conventional montage,” small-anode in the “side montage,” and small-anode in the “sham montage.” Pairwise *post hoc* comparisons were carried out using *t*-statistics with Bonferroni correction (“multcompare” in Matlab). Alpha was set at *P* < 0.05.

## Results

**FIGURE 2:**
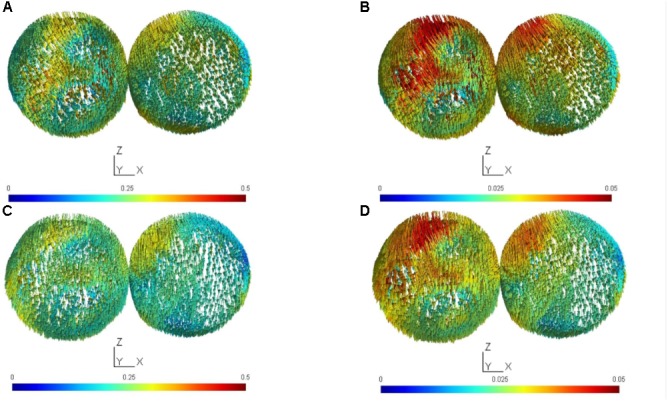
The electric field (EF) vector was computed for the targeted (targ) cortical tissue cluster found after Boolean intersection with the sphere of a radius of 1 cm centered at (–7 mm, –38 mm, 75 mm) in MNI coordinates, and the contralateral (contra) sphere with a radius of 1 cm centered at (6 mm, –38 mm, 75 mm) in MNI coordinates. **(A)** target and contralateral clusters for anodal tDCS with the large-anode, 5 cm × 7 cm at 2 mA and the cathode placed at Fp2 (10–10 EEG system), **(B)** target and contralateral clusters for anodal tDCS with the small-anode, 3.5 cm × 1 cm at 0.2 mA and the cathode placed at Fp2 (10–10 EEG system), **(C)** target and contralateral clusters for anodal tDCS with the large-anode, 5 cm × 7 cm at 2 mA and the cathode placed at T7 (10–10 EEG system), **(D)** target and contralateral clusters for anodal tDCS with the small-anode, 3.5 cm × 1 cm at 0.2 mA and the cathode placed at T7 (10–10 EEG system). Scales of EF magnitudes are different for the small and large electrodes to make it possible to identify the distribution of field magnitudes for both electrode sizes.

**FIGURE 3:**
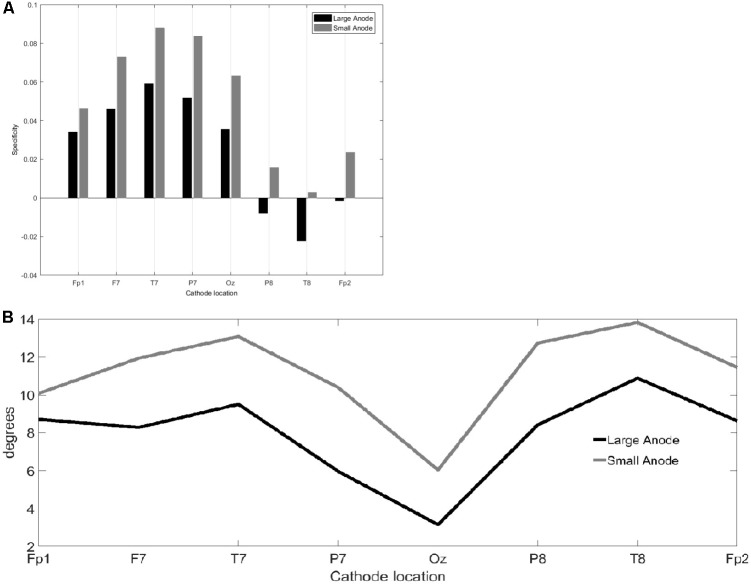
Results from the finite element modeling of the large-anode (5 cm × 7 cm at 2 mA) and the small-anode (3.5 cm × 1 cm at 0.2 mA) placed at the right-leg “target-hotspot” with different cathode locations (montages), Fp1, F7, T7, P7, Oz, P8, T6, and Fp2 (10–10 EEG system). **(A)** Bar graph comparing ${Specificity}_{comp}^{montage}$ from equation 2a. **(B)** Angle, ${Angle}_{comp}^{montage}$ from Equation 2b, of $\overset{\to }{EF}$ between the right-leg targeted and the left-leg contralateral motor volumes.

The results from the computational modeling of the electric field at the targeted right-leg motor volume [centroid at (-7 mm, -38 mm, 75 mm) in MNI coordinates] and the contralateral left-leg motor volume [centroid at (6 mm, -38 mm, 75 mm) in MNI coordinates] are shown in **Figure 2**. The maximum electric field magnitude at the targeted leg motor volume for the large-anode, 5 cm × 7 cm, at 2 mA, was around 0.4 V/m, while for the small-anode, 3.5 cm × 1 cm at 0.2 mA, it was around 0.05 V/m. Therefore, the maximum electric field strength was about one-tenth at the targeted right-leg motor volume with the small-anode (**Figures 2B,D**) when compared to that for the large-anode (**Figures 2A,C**). For the small-anode, the maximum electric field strength was found to be higher at the targeted right-leg motor volume than the contralateral left-leg motor volume with the cathode at T7 (**Figure 2D**) when compared to the cathode at Fp2 (**Figure 2B**). This difference in the electric field strength was captured with the specificity metric from finite element analysis. The ${Specificity}_{comp}^{montage}$ for the large-anode (in black) and small-anode (in gray) for different cathode locations is shown in **Figure 3A**. The T7 cathode location provided the best specificity for both the large-anode and the small-anode. This best montage identified by computational analysis with the small-anode positioned over the “target-hotspot” and the cathode over T7 was labeled “side montage” for neurophysiological testing. Also, the angle between the average electric field direction (unit vector) at the targeted right-leg and the contralateral left-leg motor volume, ${Angle}_{comp}^{montage}$, was compared, and the results are shown in **Figure 3B**. The small-anode resulted in a larger ${Angle}_{comp}^{montage}$ when compared to the large-anode, however the distribution across cathode locations, Fp1, F7, T7, P7, Oz, P8, T6, and Fp2 (10–10 EEG system) was similar for the small-anode and the large-anode.

For neurophysiological evaluation based on ${Specificity}_{neurophys}^{montage}=\frac{\left({MEP}_{\mathrm{targeted}}-{MEP}_{contralateral}\right)}{\left({MEP}_{\mathrm{targeted}}+{MEP}_{contralateral}\right)}$, the “side montage” was compared with the “conventional montage.”

**FIGURE 4:**
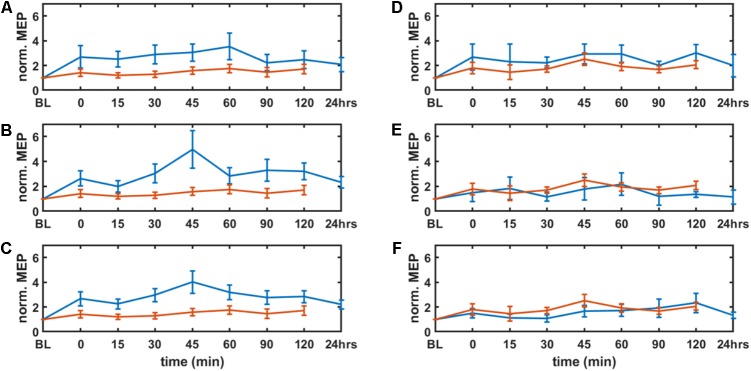
Results from neurophysiological testing – corticospinal excitability alterations evaluated by motor evoked potentials (MEP) from tibialis anterior muscles (left panel: targeted right leg, right panel: contralateral left leg) before and immediately after the completion of tDCS as well as every 15 min for the next 60 min, then every 30 min for the next 60 min for each session, and 24 h for the real stimulation conditions. MEPs were individually normalized to baseline (BL). Solid blue lines show the means of the real tDCS sessions and the solid red lines show the means of the sham tDCS sessions. The error bars show the standard error of means. The parameter space consisted of anode size (large: 5 cm × 7 cm at 2 mA and small: 3.5 cm × 1 cm at 0.2 mA), and cathode locations (10–10 EEG system: “side montage” – cathode at T7 and “conventional montage” – cathode at Fp2). **A**,**D** show the results of the large-anode “conventional montage,” **B**,**E** for the small-anode “conventional montage,” and **C,F** show results for the small-anode “side montage.”

**Figure 4** shows the results from the neurophysiological testing of corticospinal excitability changes following anodal tDCS. All results are displayed as mean ± standard error of means. The corticospinal excitability changes are presented as MEPs individually normalized to baseline (baseline-normalized MEP) from the targeted right-leg and the contralateral left-leg TA muscles before and immediately after the completion of anodal tDCS as well as every 15 min for the next 60 min, and then every 30 min for the next 60 min for each session. The repeated measure two-way ANOVA [within subject factors: time post-tDCS(min): 0, 15, 30, 45, 60, 90, 120, and tDCS-condition: large-anode “conventional montage,” small-anode “conventional montage,” small-anode “side montage,” small-anode “sham montage”] conducted for the dependent variable baseline-normalized MEP of the right-leg “target-hotspot” showed significant main effects of time post-tDCS [*F*(6) = 4.65, *P* < 0.05] and tDCS-condition [*F*(3) = 23.44, *P* < 0.05], but no significant interaction [*F*(18) = 1.18, *P* = 0.264]. For the dependent variable baseline-normalized MEP of the left-leg “contralateral-hotspot,” a significant main effect was found only for tDCS-condition [F(3) = 9.79, P < 0.05] but not for time [*F*(6) = 2.08, *P* = 0.0528] or the respective interaction [*F*(18) = 0.6, *P* = 0.9011].

**FIGURE 5:**
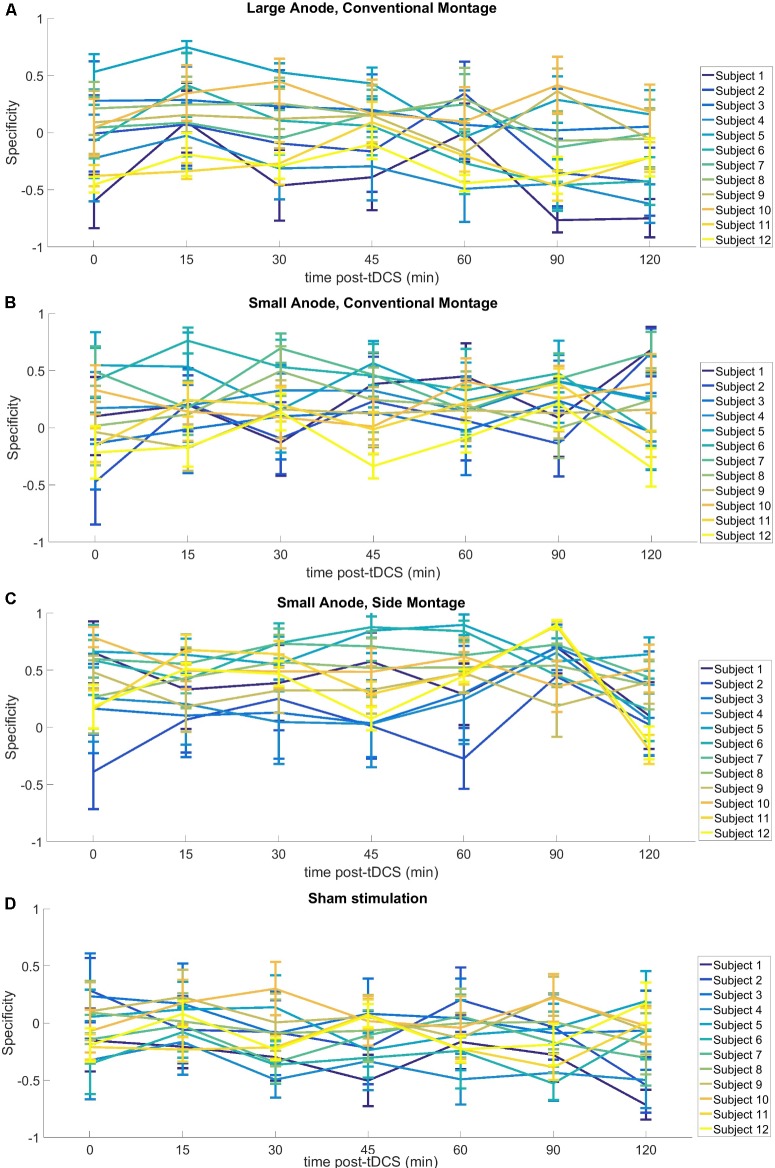
Results from neurophysiological testing – ${Specificity}_{neurophys}^{montage}$ – of the corticospinal excitability after-effects for different tDCS-conditions with regard to interindividual variability, **(A)** large-anode, “conventional montage”: large anode, 5 cm × 7 cm at 2 mA, over the right-leg “target-hotspot” with the cathode over Fp2 (10–10 EEG system), **(B)** small-anode, “conventional montage”: small anode, 3.5 cm × 1 cm at 0.2 mA, over right-leg “target-hotspot” with the cathode over Fp2 (10–10 EEG system), **(C)** small anode, “side montage”: small-anode, 3.5 cm × 1 cm at 0.2 mA, over right-leg “target-hotspot” with the cathode over T7 (10–10 EEG system), **(D)** Sham stimulation: large anode, 5 cm × 7 cm at 2 mA, at right-leg “target-hotspot” with the cathode at Fp2 (10–10 EEG system). Here, relatively large individual variability is notable, including the sham montage.

**FIGURE 6:**
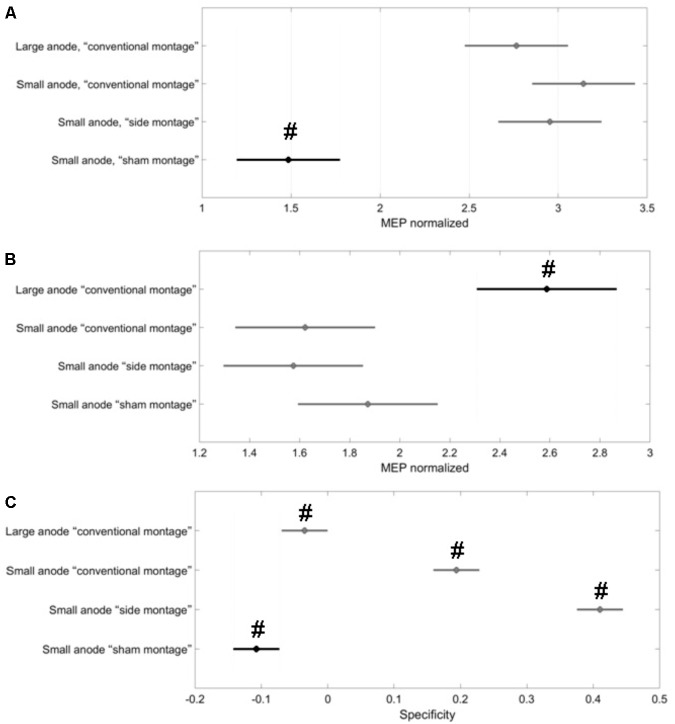
Results from the *post hoc* tests (*P* < 0.05) presented with 95% confidence intervals generated from all MEPs. Black represents the data contrasted in the respective *post hoc* comparison with the other conditions (in gray). Here, two group means are significantly different if their intervals are disjoint; they do not differ significantly if their intervals overlap. **(A)** The baseline-normalized MEP of the right-leg “target-hotspot” was lowest and significantly different (marked with #) in the “sham montage” when compared to all real tDCS conditions. **(B)** the baseline-normalized MEP of the left-leg “contralateral-hotspot” was largest for the large-anode “conventional montage,” which differed significantly (marked with #) from all other tDCS-conditions. **(C)** ${Specificity}_{electrophys}^{montage}$ values were significantly different from each other for all tDCS-conditions (marked with #), with the small-anode “side montage” having the highest mean, then the small-anode “conventional montage,” followed by large-anode “conventional montage” and then, the small-anode “sham montage.”

The ${Specificity}_{electrophys}^{montage}$ of the corticospinal excitability after-effects for different tDCS conditions is shown in **Figure 5** for the single subject level. All results are displayed as mean ± standard error of means. The *post hoc* tests using *t*-statistics with Bonferroni correction revealed that the baseline-normalized MEP of the right-leg “target-hotspot” for the small-anode in the “sham montage” was lowest and differed significantly (*P* < 0.05) from the other tDCS-conditions after intervention (**Figure 6A**). The baseline-normalized MEP of the left-leg “contralateral-hotspot” were highest for the large-anode “conventional montage,” and differed significantly (*P* < 0.05) from the other tDCS-conditions (**Figure 6B**). Consequently, ${Specificity}_{neurophys}^{montage}$ of the corticospinal excitability after-effects, which is the normalized difference between the baseline-normalized MEP of the right-leg “target-hotspot” and the left-leg “contralateral-hotspot” was found to be negative (95% confidence interval) for the large-anode “conventional montage” in the *post hoc* tests (see **Figure 6C**). *Post hoc* tests revealed that the ${Specificity}_{neurophys}^{montage}$ was significantly different (*P* < 0.05) for different tDCS-conditions, with the small-anode “side montage” having the highest mean (i.e., best montage based on ${Specificity}_{neurophys}^{montage}$), followed by the small-anode “conventional montage,” the large-anode “conventional montage,” and then the small-anode “sham montage” – see **Figure 6C**.

## Discussion

The results of this study supply information about the effects of electrode montage and anode size on the specificity of anodal tDCS after-effects on the leg motor area. All active stimulation conditions induced the expected target motor cortex excitability enhancements. Hereby, the small-anode “side montage” configuration, i.e., 3.5 cm × 1 cm anode placed over the right-leg motor “target-hotspot” with the cathode placed over T7 (10–10 EEG system) was found to be superior to both “conventional montages” with the cathode positioned over Fp2 (10–10 EEG system) in terms of specificity in both the computational analysis (**Figure 3**) and neurophysiological testing (**Figure 6C**). The simulated maximum electric field strength was about one-tenth at the targeted right-leg motor volume with the small-anode (**Figures 2B,D**), as compared to that induced by the large-anode (**Figures 2A,C**). Nevertheless, the small-anode montage altered cortical excitability, in agreement with prior works (Madhavan and Stinear, 2010), in both the “conventional montage” (**Figures 4B,E**) as well as “side montage” (**Figures 4C,F**). Therefore, the physiological effects over this target region did not correlate linearly with simulated electrical field (EF) strength. This finding is in accordance with those of a recent study, where it was shown that for a relatively large range of stimulation intensities, anodal tDCS over the motor cortex resulted in similar MEP alterations (Jamil et al., 2017), thus physiological effects may not scale linearly with electric field strength. Alternatively, it cannot be ruled out that the currently available models do not deliver sufficiently correct simulations of EF strength.

In this study, in contrast to the large-anode “conventional montage,” the small-anode electrode arrangements resulted in a positive ${Specificity}_{comp}^{montage}$ in the computational analysis for the “conventional montage” as well as the “side montage,” which was confirmed by neurophysiological testing of the ${Specificity}_{comp}^{montage}$. Here, ${Specificity}_{comp}^{montage}$ was defined based on the volume-averaged magnitude of the electric field or volume-averaged electric field strength (Opitz et al., 2015). Neurophysiological testing confirmed in concurrence with the computational analysis that the small-anode “side montage” provided the best specificity across all evaluated tDCS-conditions: large-anode “conventional montage,” small-anode “conventional montage,” small-anode “side montage,” and small-anode “sham montage.” Beyond EF strength that was used to define ${Specificity}_{comp}^{montage}$, directionality of the current flow might have relevantly contributed to the specificity differences between electrode arrangements. We found from **Figure 3B** that tDCS cathode locations over F7, T7, and P7, with the anode over the left primary motor cortex resulted in $\overset{\to }{EF}$ that was primarily in the right-to-left direction in the coronal plane at the right-leg “target-hotspot.” This is in accordance with the respective TMS results (Priori et al., 1993) showing that the threshold is lowest for MEPs in the right-leg TA muscle when the current in the TMS coil flows from the left to the right side in the coronal plane, i.e., right-to-left oriented induced current in the right-leg “target-hotspot.” **Figure 3B** shows that the EF direction differs on an average by 11.5° for the small anode and 9° for the large anode for the targeted and contralateral ROIs of the “conventional montage” (Fp2 cathode location). Here, the electric field direction is primarily posterior–anterior (PA) (rather than medio-lateral) in the “conventional montage” (Fp2 cathode location). Neurophysiological results showed that MEPs increased comparably in all real tDCS conditions for the targeted leg when compared to sham (see **Figure 6A** for the targeted leg). However, for the contralateral leg, only the large anode in the conventional montage resulted in a significant increase of MEP as compared to sham stimulation, as shown in **Figure 6B**. The relatively small difference in EF directions and higher magnitude of the electric field in the large anode conventional montage design, which covers a large volume of the brain including contralateral M1 (caused by the distant anterior position of the return electrode in the “conventional montage”) can explain the identically directed effects of stimulation at the targeted and contralateral M1 in this condition. The relatively high magnitude of the EF in this condition (∼0.4 V/m with the large anode – see **Figure 2**) should be sufficient to affect M1 bilaterally. The relatively small difference of EF directions in the right and left motor cortices, most probably caused by the long-distant *anterior* position of the return electrodes, explains the identically directed effects of stimulation with the large electrodes on both areas, taking also into account that tDCS does not have an effect only on pyramidal neurons, but also on interneurons, which might be directed relevantly in AP/PA directions (Nitsche et al., 2005). The differences between the results of the small and large electrodes with the Fp2 return electrode positions, which resulted in similarly oriented EF vectors, and roughly comparable ipsi- and contralateral EF strength, are most probably caused by different specificity values, as shown by the results of the modeling, where the small electrode resulted in higher specificity in favor of the targeted motor cortex as compared to the large electrode, which resulted in zero specificity. Moreover, the lower absolute EF strength generated by the small electrode according to the modeling results, might have contributed, taking into account that a critical EF strength is assumed to exist, below which no excitability alterations are expected. The specific foundations for these results should, however, be further explored in future studies.

Some limitations of the study should be taken into account. The SimNIBS automated software pipeline (Windhoff et al., 2013) used in this study for computational modeling did not use a subject-specific head model. Therefore, the accuracy of the computed values is limited by the dimensions, the tissues modeled, and the isotropic conductivity values selected for the volume conduction head model. Thus whereas relations between different electrode configurations and placements should be relatively reliable, exact numerical results should be treated with caution. Nevertheless, such simple head models may increase our understanding of how stimulation parameters affect the electric field distribution. For example, Faria et al. (2011) showed that the magnitude of the current density falls more rapidly for smaller electrodes so one will need a higher current density at the electrode to get the same current density (or electric field strength) at deeper cortical targets. In addition, in the “sham montage,” we observed an enhancement of post MEP amplitudes, most probably caused by difficulties of the participants to remain completely relaxed regarding muscle tone over the prolonged time course of the experiment. This most likely also resulted in high inter-individual variability in MEP measures (**Figure 5**). Another factor which might have contributed to this variability is the substantial intrinsic trial-to-trial amplitude variability of MEPs, due to state differences of brain activity, and other factors. The recently introduced EEG-adapted stimulation protocols might be helpful to reduce such variability in future (Zrenner et al., 2018). However, the variability of the MEP difference between the targeted and the contralateral RoIs (i.e., the specificity) was not affected as much (as shown in **Figure 6C**). Nevertheless, the negative specificity in the “sham montage,” in **Figure 6C**, is notable with the non-dominant leg showing higher cortical excitability alterations than the dominant leg. This asymmetry might be related to an impact of foot dominance on MEP, similar to results shown for hand dominance in young adults (Bernard and Seidler, 2012). Since only one montage was tested as sham condition, and post-tDCS measures were covering a shorter time course in the sham as in the real stimulation conditions, blinding might potentially have been compromised in some participants; however, the respective multiple-session experimental design and the randomized order of experiments should have prevented unblinding in most participants.

The results of this study might be relevant for presumptive clinical applications of tDCS for reducing post-stroke maladaptive plasticity at the unaffected contralesional hemisphere that produces inter-hemispheric inhibition (Jones, 2017). While, however, higher specificity of stimulation might be achieved relatively easily in non-lesioned brains via modeling of a standard head, and small electrode sizes might be helpful, this does not easily transfer to patients with brain lesions, in which representations of brain functions, and also physical properties of conductivities, might differ. Here, patient-specific individual head-models may be important to optimize tDCS of the leg motor area to make it a viable clinical option in post-stroke neurorehabilitation (Otal et al., 2016).

## Conclusion

We conclude that electrode size, cathodal return electrode position have a relevant impact on anodal tDCS effects on excitability of the lower limb motor cortex. In the “conventional montage” condition, the large-anode affected both the targeted and the contralateral leg motor representations in a similar way, while the small-anode in both the “conventional montage” and the “side montage” primarily affected the targeted leg motor representation in terms of corticospinal excitability alterations. Here the “side montage” resulted in more specific effects. The results of this study show that modeling in combination with physiological testing is suited to optimize tDCS protocols, and might be relevant for future studies targeting the lower limb motor cortex.

## Author Contributions

MN and AD contributed to the conception of this investigation. ÁF substantially contributed to the analysis of the electrophysiological data. ZR substantially contributed to the analysis of the computational data. AD, ÁF, WP, and MN contributed to the interpretation of the results and writing and reviewing of the manuscript.

## Conflict of Interest Statement

The authors declare that the research was conducted in the absence of any commercial or financial relationships that could be construed as a potential conflict of interest.

## Footnotes

1 www.simnibs.org

2 http://gmsh.info/
